# Phenotyping of Plant Biomass and Performance Traits Using Remote Sensing Techniques in Pea (*Pisum sativum*, L.)

**DOI:** 10.3390/s19092031

**Published:** 2019-04-30

**Authors:** Juan José Quirós Vargas, Chongyuan Zhang, Jamin A. Smitchger, Rebecca J. McGee, Sindhuja Sankaran

**Affiliations:** 1 Department of Biological Systems Engineering, Washington State University, Pullman, WA 99164, USA; j.quirosvargas@wsu.edu (J.J.Q.V.); chongyuan.zhang@wsu.edu (C.Z.); 2 Department of Crop and Soil Sciences, Washington State University, Pullman, WA 99164-6434, USA; jsmitchger@wsu.edu; 3 USDA-ARS, Grain Legume Genetics and Physiology Research Unit, Pullman, WA 99164-6434, USA; rjmcgee@wsu.edu

**Keywords:** crop monitoring, prediction model, satellite imagery, vegetation indices, crop surface model

## Abstract

Field pea cultivars are constantly improved through breeding programs to enhance biotic and abiotic stress tolerance and increase seed yield potential. In pea breeding, the Above Ground Biomass (AGBM) is assessed due to its influence on seed yield, canopy closure, and weed suppression. It is also the primary yield component for peas used as a cover crop and/or grazing. Measuring AGBM is destructive and labor-intensive process. Sensor-based phenotyping of such traits can greatly enhance crop breeding efficiency. In this research, high resolution RGB and multispectral images acquired with unmanned aerial systems were used to assess phenotypes in spring and winter pea breeding plots. The Green Red Vegetation Index (GRVI), Normalized Difference Vegetation Index (NDVI), Normalized Difference Red Edge Index (NDRE), plot volume, canopy height, and canopy coverage were extracted from RGB and multispectral information at five imaging times (between 365 to 1948 accumulated degree days/ADD after 1 May) in four winter field pea experiments and at three imaging times (between 1231 to 1648 ADD) in one spring field pea experiment. The image features were compared to ground-truth data including AGBM, lodging, leaf type, days to 50% flowering, days to physiological maturity, number of the first reproductive node, and seed yield. In two of the winter pea experiments, a strong correlation between image features and seed yield was observed at 1268 ADD (flowering). An increase in correlation between image features with the phenological traits such as days to 50% flowering and days to physiological maturity was observed at about 1725 ADD in these winter pea experiments. In the spring pea experiment, the plot volume estimated from images was highly correlated with ground truth canopy height (*r* = 0.83) at 1231 ADD. In two other winter pea experiments and the spring pea experiment, the GRVI and NDVI features were significantly correlated with AGBM at flowering. When selected image features were used to develop a least absolute shrinkage and selection operator model for AGBM estimation, the correlation coefficient between the actual and predicted AGBM was 0.60 and 0.84 in the winter and spring pea experiments, respectively. A SPOT-6 satellite image (1.5 m resolution) was also evaluated for its applicability to assess biomass and seed yield. The image features extracted from satellite imagery showed significant correlation with seed yield in two winter field pea experiments, however, the trend was not consistent. In summary, the study supports the potential of using unmanned aerial system-based imaging techniques to estimate biomass and crop performance in pea breeding programs.

## 1. Introduction

Pea (*Pisum sativum* L.) is an important source of protein in many countries and cultures [1]. Field peas have been improved through breeding programs [2] to enhance biotic and abiotic stress tolerance, increase seed yield potential, and improve nutritional quality. Traits such as days to flower, days to physiological maturity, the number of the first reproductive node [3], seed yield, and canopy volume are often measured or estimated during field phenotyping. In addition, traits such as the Above Ground Biomass (AGBM) are also assessed due to its influence on seed yield, canopy closure, and weed suppression. It is also the primary yield component for peas used as a cover crop and/or forage crop.

Conventional plant phenotyping methods based on field observations and manual data collection can be time consuming and result in measurement errors. Using remote sensing tools, small to large breeding trials can be mapped with higher temporal and spatial homogeneity on the field-sampled data [4]. With the wide range of Unmanned Aerial Systems (UASs) and sensors currently available, many crops can be phenotyped more efficiently. For example, wheat ear density estimates using a digital red-green-blue (RGB) camera were similar to estimates made using traditional methodologies [5]. Cameras integrating RGB and near infrared (NIR) sensors have been used to generate vegetation indices (VIs) such as Normalized Difference Vegetation Index (NDVI) [6,7,8] and Normalized Difference Red Edge (NDRE) to assess crop status [9,10,11]. The combination of RGB bands to compute Green-Red Vegetation Index (GRVI) can also be used for biomass estimates [12,13], yield monitoring [14], and canopy volume estimates [15]. These VIs can also be used to phenotype in a high-throughput manner [16]. Crop biomass has been found to be correlated with NDVI [17,18,19], and has been used to build prediction models using machine learning approaches such as support vector machine [20,21], regression models [22], random forest [23], artificial neural networks [24], and least absolute shrinkage and selection operator (Lasso) [25]. Nevertheless, much of this work on crop biomass estimation has only been done in small grains such as barley and forest tree species. 

The 3D reconstruction of field plots is also possible based on stereo vision high density data collected during UAS missions. Such 3D information can be used to generate Digital Surface Models (DSM) with elevation data in m above mean sea level. The Crop Surface Model (CSM) is computed by extracting the terrain topography, using the Digital Terrain Model (DTM), from the DSM. The CSM utilizes ground elevation as a reference, thus providing object information Above Ground Level (AGL) such as canopy height [26]. In spite of the recent developments in the UAS-boarded geo-location devices, without a Real Time Kinematics (RTK) module on the UAS, the position may oscillate several meters in radius. Due to the high cost of the UAS-RTK platforms, an alternative solution to increase the elevation data accuracy is to use Ground Control Points (GCP) measured with RTK field devices [27]. Highly accurate RTK-corrected data can be used for canopy height measurements, crop volume [28], biomass [12,13], and crop lodging [26].

Satellite-based remote sensing data also plays a key role in large scale crop monitoring [29], yield forecasting [30], crop damage assessments [31], crop disease and pest distribution monitoring [32], irrigation requirement estimations [33], and site-specific management practices [34]. In spite of its advantages, the use of satellite-based imagery for plant phenotyping has been limited due to its generally low temporal and spatial resolution. However, recent developments in satellite imagery can provide sub-meter multispectral imagery with revisit times of less than 5 days. Theoretically, an image with pixel dimensions lower than the plot length and width can be expected to provide similar information as UAS data.

Remote sensing methods have been used to phenotype plant height and biomass [35] in crops including sorghum, barley, and rice with coefficients of determination between ground data (physical biomass) and VIs of 0.63–0.84, depending on the growth stages and crop types [12,13]. However, in pea breeding programs, the methods need to be evaluated for feasibility and accuracy. With this goal, the overall objective in this study was to determine the reliability of utilizing UAS-based image data (GRVI, NDVI, NDRE, plot volume, canopy height, and canopy coverage) in determining phenological and agronomic plant traits in winter and spring pea-breeding programs. In addition, a comparison between UAS-based and medium-resolution satellite image data with biomass and seed yield was performed to determine the viability of using orbital imagery data for field plant phenotyping.

## 2. Materials and Methods

### 2.1. Study Area

**Figure 1:**
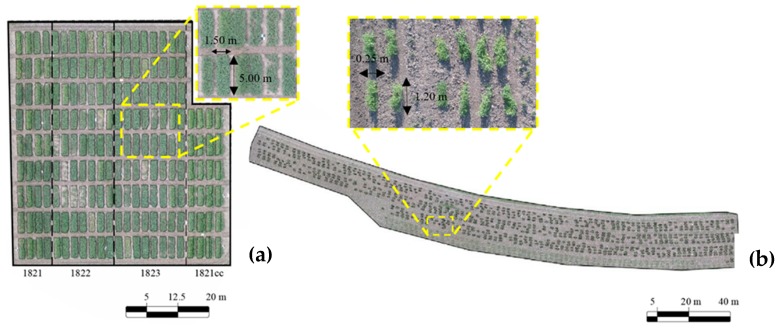
Experiments and plot dimensions for (**a**) winter and (**b**) spring field pea sites.

The winter pea field experiments were located at the Washington State University’s Spillman Agronomy Farm near Pullman, Washington, USA (46°41′54.71″ N; 117° 8′45.22″ W). Data were collected at 365, 784, 1268, 1725, and 1948 accumulated degree days (ADD), corresponding to 15 May, 30 May, 19 June, 5 July, and 16 July 2018, respectively. ADD were calculated at a 0 °C base temperature [36] from 1 May 2018. The winter pea experiments from the United States Department of Agriculture (USDA) Agricultural Research Service winter pea breeding program were: 1821 (Austrian Winter Pea Advanced Yield Trial), 1821cc (Cover Crop Winter Pea Advanced Yield Trial), 1822 (Food Quality Winter Pea Advanced Yield Trial), and 1823 (Food Quality Winter Pea Preliminary Yield Trial). The experimental design of each trial was a randomized complete block design with three replicates. Experiments 1821cc, 1821, 1822, and 1823 had 5, 10, 20, and 20 entries, respectively. The plot size was approximately 1.5 m × 5.0 m (Figure 1a). The planting date was on 11 October 2017, and seedlings emerged 15 to 30 days later. In all the winter pea experiments data collected included days to 50% flowering (F50), leaf type: normal (*Af*) or semi-leafless (*af*), days to physiological maturity (PM), number of the first flowering node (FN), and seed yield (SY); additionally, in experiments 1821 and 1821cc, AGBM data were collected at flowering (1268 ADD). Flowering, an important trait evaluated in breeding programs, refers to the appearance of reproductively receptive flowers on plants. During this time, pollen is transferred to the stigma, the ovules are fertilized, and seed development commences. A plot is ‘flowering’ when 50% of the plants have flowers that are at anthesis. For AGBM estimation, 50% of each plot was harvested and fresh weight was measured. In order to evaluate the accuracy of the DSMs, at 1268 ADD ground truth canopy height (CH_GT_) measurements were taken from 18 randomly selected plots (3 plants/plot) within the field area of winter pea experiments.

The spring pea field was located in the Plant Materials Center of the USDA, Washington, USA (46°43′12.83″ N; 117° 8′33.88″ W). The plant materials in this experiment were the USDA Pea Single Plant Derived Core Collection (PSP), a genome wide association mapping population that has been previously phenotyped and genotyped [37]. The 307 accessions were planted in a randomized complete block design with three replications. This experiment was planted on 14 May 2018, plots consisted of two, 1.2 m long rows (Figure 1b). Once the plants reached 50% flowering, CH_GT_, lodging, and leaf type were measured, and AGBM (as total dry weight) was assessed through destructive sampling of the entire plot. Lodging was measured as the ratio of the height of the canopy divided by the total length of the plant, i.e., the closer the ratio is to 1.0, the more erect (less lodged) the plants are. Data collection occurred on 28 June (1231 ADD), 5 July (1424 ADD), and 12 July (1648 ADD). ADD was calculated from the planting date. 

### 2.2. UAS Data Collection

In the winter and spring pea experiments, a total of 10 GCPs were uniformly distributed over each experimental area, including the field edges to minimize the planimetry error [38]. A marker stake was placed at each GCP location and remained in place throughout the season. Prior to each flight, boards (0.8 m × 0.5 m) that could be seen in the resulting UAS images were placed at each GCP position. The coordinates of each point were recorded at the end of the experiment with a RTK system based on SPS850 Global Navigation Satellite System receivers from Trimble Inc. (California, USA), which integrates a 450–900 MHz transmitter/receiver radio and a 72-channel L1/L2/L2C/L5/GLONASS GPS receiver.

RGB data was collected with a DJI-Phantom 4 Pro (Shenzhen, China) using its original 20 MP resolution, 25.4 mm CMOS camera with lens characteristics of 84° field of view and 8.8 mm/24 mm (35 mm format equivalent). DJI-phantom 4 Pro is powered with 6000 mAh LiPo 2S battery and the speed during data acquisition was 2 m/s; it works with the Global Navigation Satellite System (GNSS: GPS and GLONASS constellations) with average horizontal and vertical accuracies of ~0.5 m and ~1.5 m, respectively. The high-density data were collected in a double grid pattern with 90% overlap (both directions) at 20 m AGL (0.005 m of ground sample distance/GSD) to generate high accuracy digital surface models. As high-density images were collected from different angles (more points of view for each object on the field), it was expected that the process would improve the quality of the 3D reconstruction. The multispectral information was captured using a Double 4K camera (Sentera LLC, Minneapolis, USA) of 59 × 40.9 × 44.5 mm dimensions with 12.3 MP (0.005 m GSD) resolution of five spectral bands. The central wavelength and full-width half maximum data for R, G, B, red edge (RE), and NIR spectral bands were 650 nm and 64 nm, 548 nm and 44 nm, 446 nm and 52 nm, 720 nm and 39 nm, and 839 and 20 nm, respectively. This sensor was mounted on an ATI-AgBOT^TM^ (ATI LLC., Oregon, USA) quadcopter with 1012 400 kv motor and dual 6000 mAh batteries; its positioning system is 3DR uBlox GPS (UAV Systems International, Las Vegas, USA) that works with a 3 V lithium rechargeable battery at 5 Hz update rate and a low noise regulator of 3.3 V. The multispectral data were collected in a single grid pattern with 80% frontal overlap and 70% side overlap, also at 20 m AGL. A white reference panel (0.25 m × 0.25 m; Spectralon Reflectance Target, CSTM-SRT-99-120) (Spectra Vista Cooperation, New York, USA) was placed on the field for radiometric correction during image processing.

### 2.3. Satellite Data Acquisition

A multispectral 1.5 m-GSD SPOT-6 satellite image was acquired from AIRBUS Defense & Space (Leiden, The Netherlands). The image captured on 3 June 2018 (close to 784 ADD) is composed by four spectral bands with the following range: R (625–695 nm), G (530–590 nm), B (455–525 nm), and NIR (760–890 nm). The original image was atmospherically corrected, but not geo-referenced. The satellite information was not used for spring pea plot evaluation for three reasons: (1) at 1.50 m GSD, it was not possible to differentiate between plots (~1.20 × 0.30 m), (2) at the time of the data capture, the plants in the spring pea trials were in early growth stages and small, and finally (3) alternative satellite images matching the UAS data collection dates were unavailable. 

### 2.4. UAS-Based Imagery Analysis

Pix4D^TM^ software was used to create the mosaics and DSM from both sensors (RGB and multispectral) through the 3D map template. During the stitching process, each RTK-GCP was fixed by identifying its position with 10 to 15 checkpoints representing GCP location on individual images (both fields and all data points). For the winter pea experiments, 5 RGB, 5 multispectral and 5 DSM mosaics were generated; while 3 RGB, 3 multispectral and 3 DSM mosaics were generated for the spring pea experiment. The white reference panel (99% reflectance in RGB-RE-NIR spectral range) imaged during each data collection was used to correct the image pixels in each band. Following this, using the “Array” command in AutoCAD (version 2018), the polygons representing each winter pea plot were digitized in a *.dxf format and further translated into ^*^.shp. As the spring pea plots did not present a uniform grid pattern, they were directly digitized in ^*^.shp format using Quantum GIS (QGIS, version 2.18.22). Each plot was labeled with plot ID based on experimental details. 

The green-red vegetation index, normalized difference vegetation index, and normalized difference red edge index were computed using the following equations.

(1) $$GRVI=\frac{\left(G-R\right)}{\left(G+R\right)}$$

(2) $$NDVI=\frac{\left(NIR-R\right)}{\left(NIR+R\right)}$$

(3) $$NDRE=\frac{\left(NIR-RE\right)}{\left(NIR+RE\right)}$$

where *R*, *G*, *RE*, and *NIR* represents the reflectance in the red, green, red edge, and near infrared bands. The *DSM* (in m above the mean sea level) was obtained from the stitched image data. To extract the *CSM*, with the canopy height (in m AGL) information, the *DTM* was created based on the interpolation of elevation data over bare soil points, and subtracted from the *DSM* (Equation (4)).

(4) CSM=DSM−DTM

**Figure 2:**
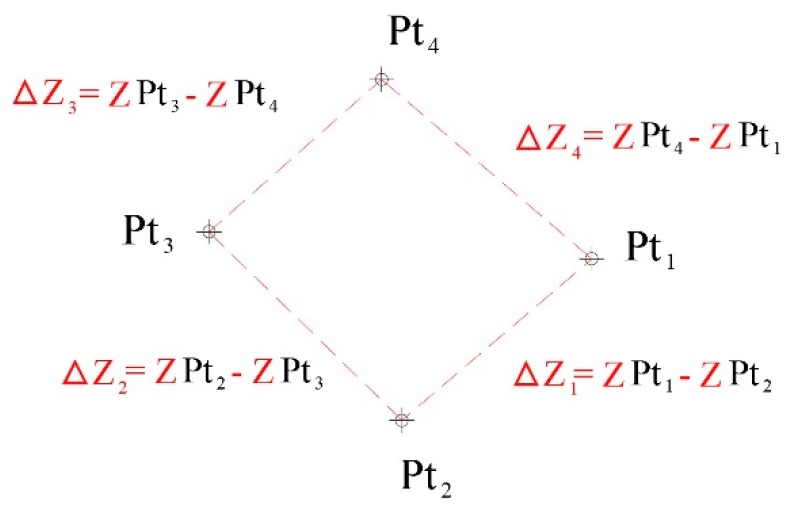
Graphical exemplification of ΔZ’s.

Using data from the winter pea field plots at 1268 ADD as reference, an assessment of the quality of the RTK geo-rectification was performed by estimating the vertical position error (VPE) and the horizontal position error (HPE) [39,40] of the rectified and non-rectified mosaic images from the two sensors (RGB and multispectral). The *HPE* was calculated using Equation (5). The VPE was estimated as the sum of the changes in elevation among adjacent points calculated with the non-rectified image (Δ*ZNR*) subtracted from those calculated with the rectified image (Δ*ZR*) (Equation (6)).

(5) $$HPE=\frac{\sqrt{EE^{2}+NE^{2}}}{n}$$

(6) $$VPE=\frac{\left(\sum_{i=1}^{n}\Delta ZR-\sum_{i=1}^{n}\Delta ZNR\right)}{n}$$

where *HPE* and *VPE* are horizontal and vertical position errors, *EE* and *NE* are East and North direction errors, Δ*ZNR* and Δ*ZR* are elevation differences from non-rectified and rectified images, and total number of samples (*n*) is 4 (Figure 2). The Δ*Z* is the sum of absolute difference in the elevation between two contiguous points (Δ*Z*_1_+Δ*Z*_2_+Δ*Z*_3_+Δ*Z*_4_, Figure 2).

From the CSM, the UAS-based CH (CH_UAS_), Canopy Coverage (CC) and Plot Volume (PV) were estimated. The CSM was segmented into two categories where pixels above 0.15 m AGL were classified as “canopy”, and pixels below 0.15 m AGL were classified as “non-target canopy” to eliminate weeds and other noises from the crop of interest. The 0.15 m was set as empirical threshold selected manually based on observations. The count of “canopy” pixels of a single plot was multiplied by the pixel area (e.g., 25 × 10^−6^ m^2^) to get the CC (m^2^). The PV (m^3^) was computed by multiplying the CH_UAS_ with CC. The binary image (non-canopy and canopy) was also used as a soil mask image. 

With the “Zonal Statistics” plugin in QGIS, the mean (as relative vigor) and sum (as absolute vigor) statistics of the three VIs, CH_UAS_, CC, and PV were extracted and recorded in the attribute table of the plot polygons, where each plot was differentiated based on its specific ID. In order to verify the consistency of the data across time, the three VIs and the CH_UAS_ were plotted as a function of the ADD and compared with a reference dry matter curve [41]. 

In addition to the features specified above, lodging assessment was performed in spring pea. The changes in the *CH_UAS_* between first and second data points, and between the first and third data points were employed to calculate the lodging in spring pea. When a plot lodges, not only does the *CH* decrease, but the *CC* increases, due to an increase in surface area. For these reasons, both features were utilized during lodging estimation. For the lodging estimation between data points 1 and 3, the difference in absolute *CC* values was multiplied with the differences between *CH_UAS_* data (Equation (7)).

(7) $$Lodging^{1-3}=\left[\left(CH_{UAS}^{1}\right)-\left(CH_{UAS}^{3}\right)\right]\times \left[\left(CC^{1}\right)-\left(CC^{3}\right)\right]$$

where 1 and 3 represent data collected at time points 1 and 3, 1231 ADD and 1648 ADD, respectively. 

Green band (from RGB orthomosaic) frequencies were plotted for the two leaf types in the spring and winter peas. Additionally, the mean and the standard deviation of the green reflectance were also computed as indicators of greenness and its variability. This processing was carried with the multispectral mosaic collect at 1231 ADD in the spring pea plots and 1268 ADD in winter pea plots. 

### 2.5. Satellite-Based Imagery Analysis

Using the “Georeferencer” tool in QGIS, the satellite image was rectified to the correct location. First, based on satellite archive Bing imagery (Bing aerial with layers) displayed with the “Open Layers” plugin, the original image was geo-located to its respective region with an error that would oscillate between 1–5 m. Second, the specific location of the winter pea experimental field was corrected to a sub-meter accuracy using the UAS RTK-mosaics as reference by matching the corner points of the field. In order to increase the resolution of the multispectral data from 6.0 m GSD to 1.5 m GSD, a pan-sharpening processing, based on a higher resolution panchromatic band, was performed in Erdas Imagine (version 14.1, Hexagon Geospatial) using the high pass filtering algorithm, which presented the clearest contrast between soil and vegetation pixels, compared with other methods like principal component analysis, hyperspectral color sharpening, and Brovey transform. GRVI and NDVI were computed with the satellite image following Equations (1) and (2). The mean and sum statistics were extracted from the plot polygons layer created for low altitude satellite imagery.

### 2.6. Statistical Analysis

Pearson’s correlation matrix between the ground truth and UAS-based data, averaged by entry, was calculated in RStudio (Version 1.1.423). For spring peas, the correlations were calculated using plot-by-plot comparisons, since the replicates of the same entry were not always harvested on the same day. Using RStudio, the least absolute shrinkage and selection operator algorithm [25] was employed to predict AGBM using the well-correlated image features. To assess the Lasso prediction accuracy, using 85% of the original dataset, a cross-validation of the mean absolute error and the correlation coefficient (*r*) between the estimated and actual values were computed. The features were centered and scaled using the ‘preProcess’ function for comparable coefficient generation, where mean data was subtracted from each value of vegetation indices and divided with standard deviation. With the winter pea data, the resulting coefficients from Lasso were used to estimate the AGBM in experiments 1822 and 1823 where ground truth AGBM data were not available. 

## 3. Results and Discussion

### 3.1. UAS-Based Position Data Accuracy

The horizontal and vertical dilutions of precision (HDOP and VDOP) representing the GCP coordinates reading accuracy are shown in Table 1. The horizontal and vertical position errors using RGB and multispectral mosaic images without RTK rectification were higher than those with RTK rectification (Table 2). 

After image rectification, the correlation coefficient between CH_GT_ and CH_UAS_ increased for both RGB- and multispectral-CSMs (Table 3). The differences between CH_GT_ and CH_UAS_ can be attributed to human error during ground truth data collection, and some variances in the grid pattern and overlap percentages, since the resolution and the flight altitudes of sensors were similar. Despite observing higher accuracy and correlation between CH_GT_ and CH_UAS_, the use of a double grid pattern and high overlap percentage may not be necessary to monitor research plots with simple geometry, such as in evaluated winter and spring pea breeding experiments. This will in-turn save battery life (thus increasing flight time and efficiency), data storage space, and image processing time. The use of the double grid pattern and higher overlap percentages with RTK-GPS rectification are necessary for monitoring and 3D mapping of more complex crop geometry, such as plant architecture with thin and narrow canopies (e.g., apple orchards and grape vineyards). Furthermore, the centimeter to sub-centimeter accuracy in the horizontal and vertical positions obtained with the RGB mosaic suggest its functionality to generate accurate plot length data, which is an important trait frequently monitored in breeding programs to estimate yield per unit area.

### 3.2. Winter Pea Growth and Development

**Figure 3:**
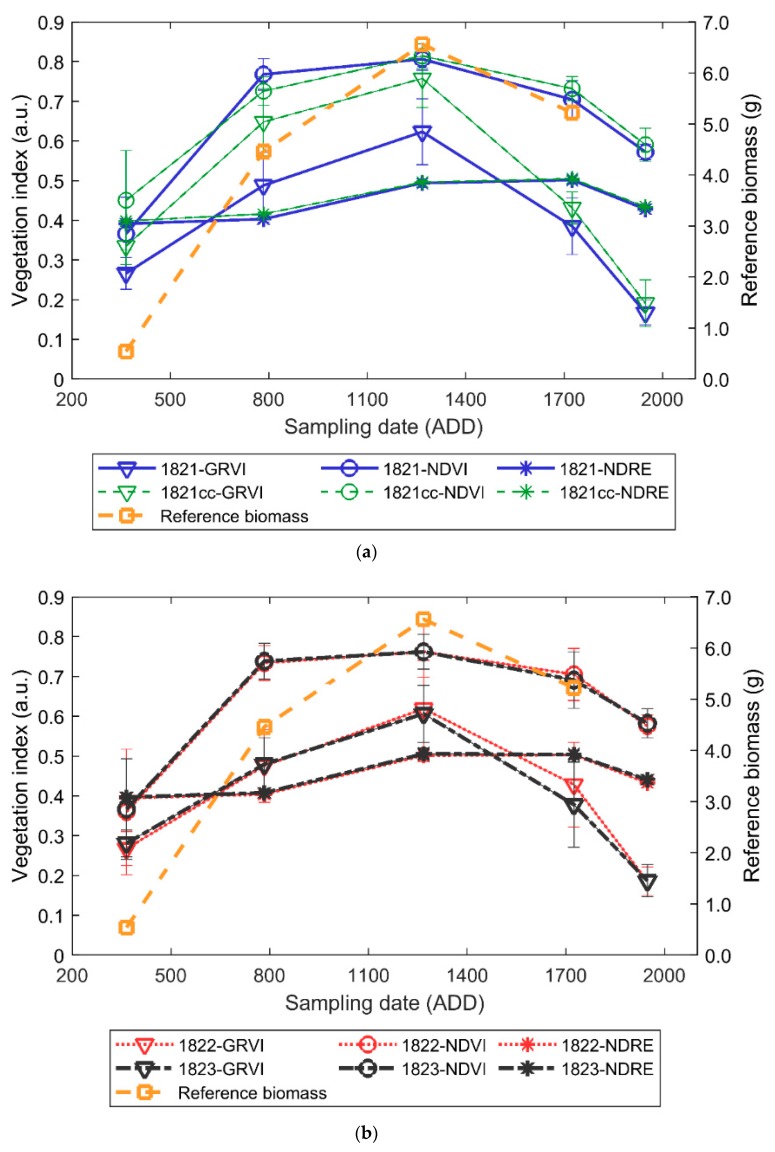
Average and standard deviation of GRVI, NDVI, and NDRE data acquired from winter field pea experiments (**a**) 1821, 1821cc, (**b**) 1822, and 1823 at different growth stages compared with a dry matter curve obtained from Reference [41].

**Figure 4:**
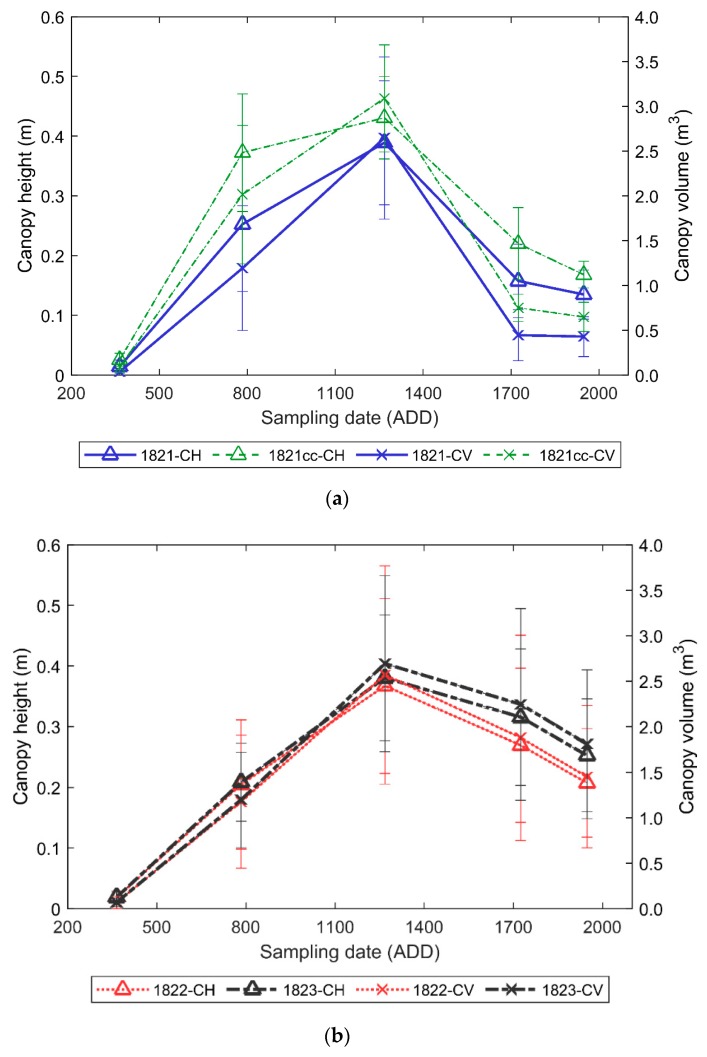
Average and standard deviation of UAS-based canopy height and canopy volume data acquired from winter field pea experiments (**a**) 1821, 1821cc, (**b**) 1822, and 1823 at different growth stages compared with a dry matter curve obtained from [41].

The average vegetation index and plot volume data across different time periods in winter pea showed a similar pattern as the reference dry matter curve [41]. At the beginning of the season, the three VIs values were about 0.30 units. At 784 ADD, the GRVI and NDVI indices increased, while the CH and PV averaged approximately 0.24 m and 0.20 m^3^, respectively (Figure 3 and Figure 4). The GRVI, CH_UAS_, and PV continued to increase until 1268 ADD; however, the NDVI changed marginally, which could be due to saturation. After 1268 ADD (flowering), the VIs decreased as the plants approached physiological maturity and senescence. Similarly, the CH_UAS_ and PV also decreased at the end of the season because of maturity and crop lodging. While a similar pattern was observed with NDVI and GRVI data, the NDRE data were low, which could be due to less abiotic stress in winter pea experiments during the season. 

The data validates the generic vegetative growth stages, where the crop canopy vigor increases and reaches maximum photosynthetic activity at flowering, resulting in a higher NIR reflection and absorption in visible wavelengths, which can be observed from the increase in VI values from 365 ADD to 1268 ADD. During the seed development and pod filling stage that represents the translocation of photo-assimilates to the seeds after flowering, there is a decrease in leaf biomass accumulation, which can be observed with a decrease of VI values. 

### 3.3. Correlation between Image Features and Performance Traits

In couple winter pea experiments, a strong correlation between image features (GRVI, NDVI, NDRE, CH_UAS_, CC, PV) and seed yield was observed, especially at 1268 ADD. At 1268 ADD, most image features were also correlated with FN (Table 4). In experiment 1823, at 1268 ADD, high correlation coefficients between image features and F50, PM, and SY were observed. In experiment 1822, high correlations between image features and SY were observed starting earlier in the season. In general, imaging between 1268 ADD (flowering) and 1725 ADD (pod development) is recommended for capturing yield differences. There were no significant differences between sum and mean vegetation index values.

The lower correlations found at early stages (365 ADD) could be attributed to the distortions caused by the brightness of bare soil within the plots [42]. To overcome this limitation, Badgley et al. [43] proposed the NIR vegetation reflectance indicator (NIRv), where 0.08 is subtracted from the product of the total NIR and NDVI, which represents the proportion of the digital number of a pixel attributed to the vegetation. In the present study, when NIRv was used, an increase in the correlations between NIRv with SY and AGBM at 365 ADD (Table 5) was observed, which did not affect relationships at 1268 ADD (high canopy cover). This suggests the importance of NIRv usage as an indicator for UAS-based ABGM predictions under high soil exposure environments or early growth stages. 

**Figure 5:**
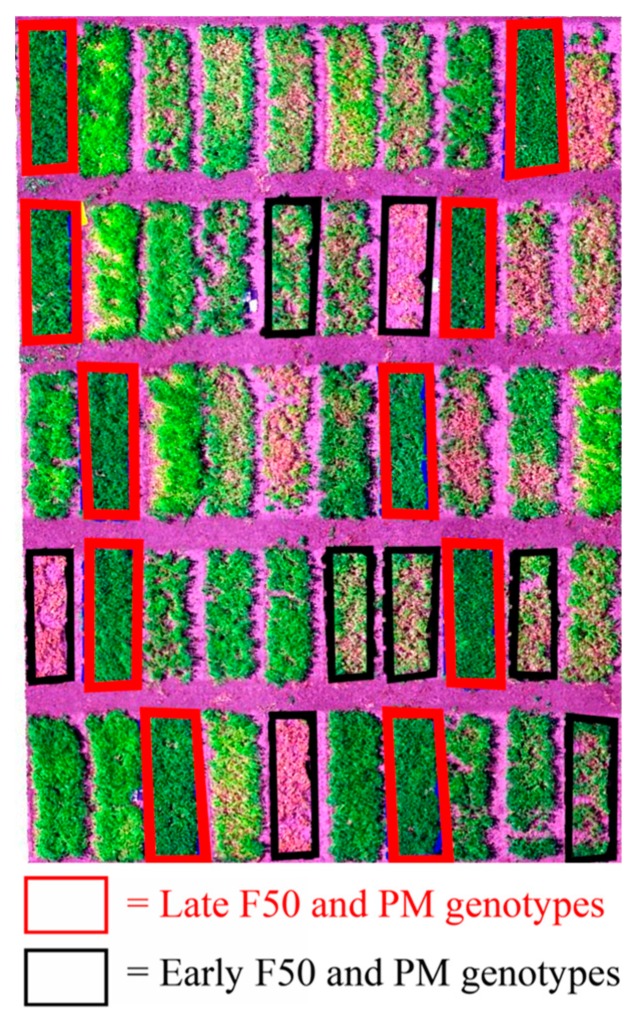
Early and late F50 and PM entries marked on RGB image acquired from experiments 1822 and 1823 at 1725 ADD.

According to the Reference [41], the high yielding genotypes have a larger leaf area index that leads to accumulation of more biomass during flowering. This is validated by the strong correlations found between leaf area index related image features such as CC and yield at 1268 ADD. Furthermore, an increase in the correlations with the phenological traits was detected in the winter pea experiments at 1725 ADD during pod development and maturity. These stronger correlations are attributed to the contrasting canopies characteristics among early and late F50 and PM plots that were easily captured with remote sensing data (Figure 5).

**Figure 6:**
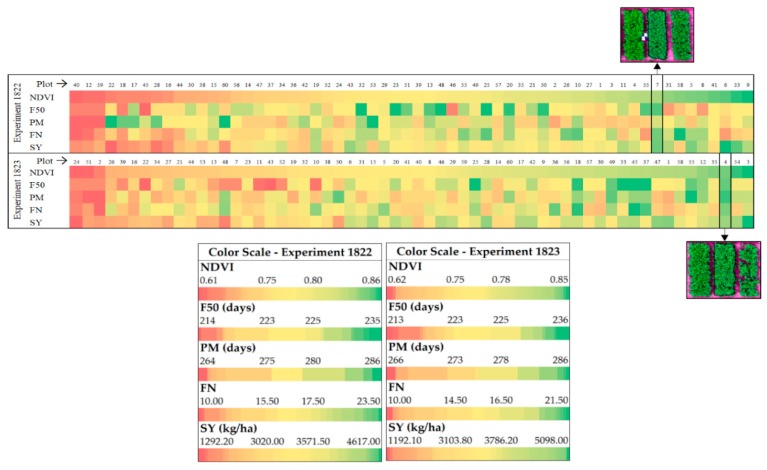
Plots of experiments 1822 and 1823 in an ascendant NDVI order, and its comparison with F50, PM, FN, and SY at 1268 ADD.

The FN is also related to the flowering time in the sense that lower FN [44] can be associated with earlier F50 and senescence in winter pea. This can explain the high correlations between FN and GRVI-mean, CH_UAS_, and PV at 1725 ADD and 1948 ADD, when image features from lower FN entries with early F50 and senescence could be differentiated from higher FN entries. Additionally, the impact of FN on yield can also be captured with VIs in winter pea, where earlier flowering entries may have more reproductive nodes per plant and therefore higher seed yield (Figure 6). 

At 1231 ADD, in the spring pea experiment, PV was significantly correlated with CH_GT_ (Table 6). The CH_UAS_, GRVI, and NDVI were correlated with CH_GT_ at 1231 and 1648 ADD. Estimations based on elevation data, such as CH_UAS_ and PV demonstrated high correlation with the CH_GT_ measurements in spring pea in most cases, which could be because these features were correlated with F50, FN, and SY as found in some winter pea experiments.

### 3.4. Correlation between Image Features and AGBM

In the winter pea experiments, strong correlation between GRVI-sum, NDVI-sum, and NDRE-sum with AGBM were found at 1268 ADD (Table 7). The correlation was lower between AGBM with NDVI and NDRE mean values. The elevation-based features were not significantly correlated to AGBM. However, in the spring pea experiments, better correlations between image features and AGBM were found, especially at 1231 ADD. The variability in F50 among the accessions in the spring pea experiment could have contributed to higher correlation with remote sensing data (Table 7). The major finding from the spring pea dataset was that the PV extracted from images was consistently correlated with AGBM, which could be useful in breeding programs.

### 3.5. AGBM Prediction with Model Development

**Figure 7:**
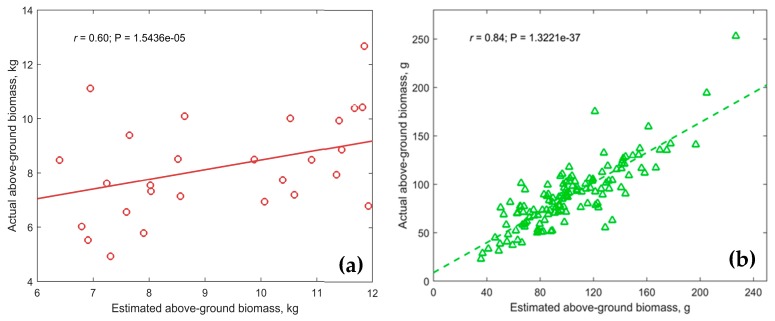
Estimated and actual AGBM correlation in the (**a**) winter and (**b**) spring field pea experiments.

With the winter pea data, the Lasso method was implemented using highly correlated image feature data at 1268 ADD to predict AGBM. The results from this model showed a R^2^ of 0.99 at F < 0.001 significance with four features. The resulting equation for AGBM estimation is defined as:

(8) $$AGBM_{Est}=\left\{\left[\left(GRVI_{Sum}*a^{1}\right)+\left(NDVI_{Sum}*b^{1}\right)+\left(NDRE_{Sum}*c^{1}\right)+\left(CC*d^{1}\right)\right]+e^{1}\right\}/1.5E^{4}$$

where *a*_1_ to *c*_1_ represent the coefficients generated by Lasso for *GRVI-sum* (*a*_1_ = 1.56), *NDVI-sum* (*b*_1_ = −0.83), *NDRE-sum* (*c*_1_ = 0.75), *CC* (*d*_1_ = −0.01), and the intercept (*e*_1_ = 8.85) of the function. To cross validate, the equation (Equation (8)) was used to estimate the AGBM in the complete data set from experiments 1821 and 1821cc at 1268 ADD. The correlation coefficient between estimated and actual AGBM was 0.60 (*P* < 0.001) (Figure 7), with a mean absolute error of 2.82 kg.

With the spring pea data, the Lasso method was implemented using the information from PV, GRVI (sum), NDRE (sum), and NDVI (sum and mean) at 1231 ADD as the best correlated scenarios. The results from the model showed a R^2^ of 0.74 at F < 0.001 significance with five features. The equation for AGBM estimation is defined as (Equation (9)):

(9) $$AGBM_{Est}=\left\{\left[\left(GRVI_{Sum}*a^{2}\right)+\left(NDVI_{Sum}*b^{2}\right)+\left(NDVI_{Mean}*c^{2}\right)+\left(NDRE_{Sum}*d^{2}\right)+\left(PV*e^{2}\right)\right]+f^{2}\right\}/1.0E^{3}$$

where *a*_2_ to *d*_2_ represent the coefficients for *GRVI-sum* (*a*_2_ = 7.80), *NDVI-sum* (*b*_2_ = 2.46), *NDVI-mean* (*c*_2_ = 4.98), *NDRE-sum* (*d*_2_= 4.98) and *PV* (*e*_2_ = 10.73), and the intercept (*f*_2_ =87.83) of the function. During validation with the complete data set at 1231 ADD, the correlation coefficient between the estimated and the actual AGBM was 0.84 (*P* < 0.001) (Figure 7) with a mean absolute error of 19.28 g.

**Figure 8:**
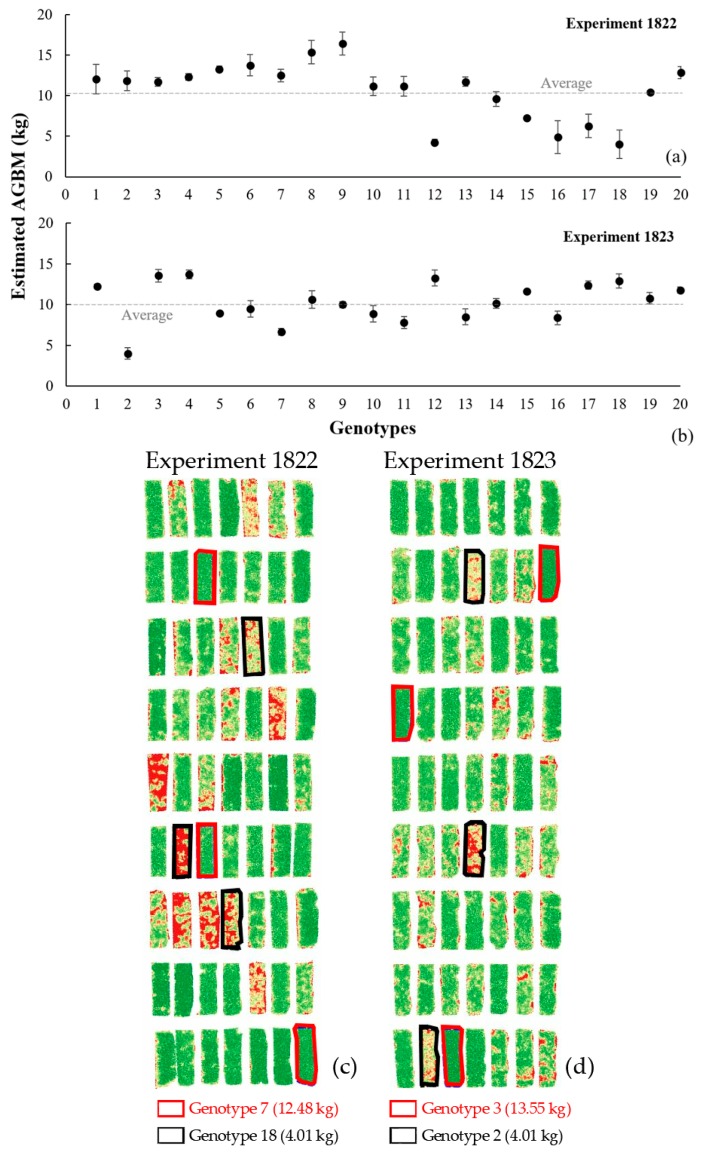
AGBM estimation for the 20 entries in experiments (**a**) 1822 and (**b**) 1823 using Lasso method with image data acquired at 1268 ADD, and (**c** and **d**) its respective NDVI maps highlighting the three replicates of the entries with the lowest (outlined in black) and highest (outlined in red) AGBM estimations.

In both pea types, sum data of GRVI, NDVI, and NDRE were variables that made a strong contribution to the AGBM estimations; alongside PV and mean NDVI in spring pea and CC in winter pea, that had an impact on the predictions. Equation (8) was used to estimate the winter pea AGBM based on 1268 ADD data. The estimated AGBM values for the 20 entries are plotted in Figure 8. The entries with the lower AGBM estimations had low canopy cover and low vegetation index values. In experiment 1822, entries 6 to 9 and 20 had AGBM estimations above the average; while, entries 12 and 14 to 18 were clearly below the average. However, in experiment 1823, the AGBM estimated for the majority of the entries were close to or below average, except for entries 1, 3, 4, 12, 15, 17, and 18. The higher AGBM accumulation entries predicted in field pea experiments shared a lower FN, F50, and PM, and higher SY. The results presented in this study need to be integrated with multiple season data in order to create a larger data-pool to build a robust machine learning prediction method.

### 3.6. Leaf Type Characterization

**Figure 9:**
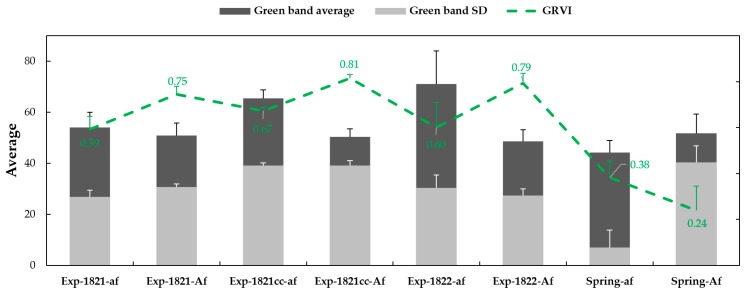
Green band average and SD, and the GRVI of *af* and *Af* leaf types at 1231 ADD and 1268 ADD in spring and winter peas.

The GRVI, and average and standard deviation of green bands from spring and winter pea field plots are presented in Figure 9. The average green reflectance data in semi-leafless entries were higher in the winter pea plots than spring pea plots, while GRVI values showed an opposite pattern. In winter pea, the variation in greenness was clearly different between experiments, which could be resulting from different pigmentation (anthocyanin), but not between leaf types. Higher variability was detected in the normal leaf type. Based on green band average, the leaf type was classified with 90%, 73%, and 87% accuracy in winter pea experiments 1821, 1821cc, and 1822, respectively, and with 74% accuracy in spring pea. 

Further investigation of leaf type characterization as well as pigmentation (chlorophyll, anthocyanin) and differences in anatomical and morphological structures is needed. The leaf type influences the total leaf area (hence biomass) and also strongly affects lodging tolerance. When the correlation analysis between GRVI-sum with AGBM was calculated by leaf type, the correlation coefficients increased (Table 8). Thus, it may be important to integrate leaf type classification with remote sensing data analysis for more robust variety selection. 

### 3.7. Lodging Estimation

**Figure 10:**
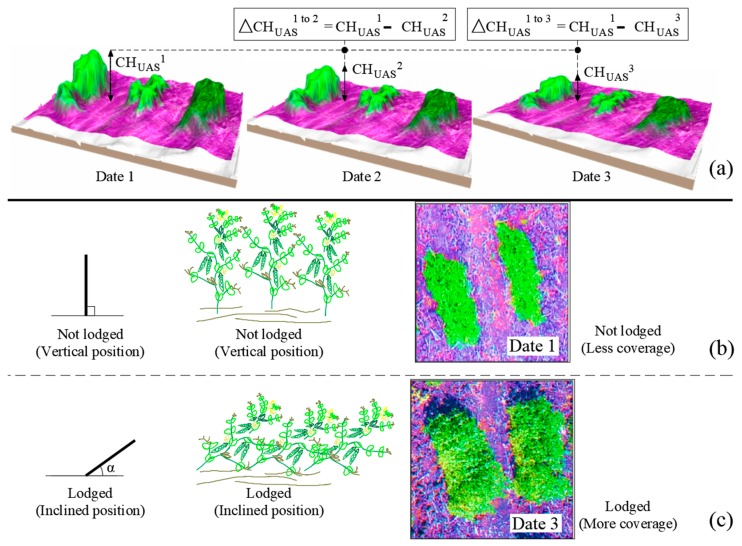
(**a**) Lodging estimation based on the differences between CH data acquired at multiple days, (**b**) sample image showing non-lodged plots with lower canopy cover, and (**c**) sample image showing lodged plots with higher canopy coverage.

Despite the small size of spring pea plants and the plots, the use of elevation data was promising for monitoring lodging. It is hypothesized that the relationships will grow stronger with larger plot sizes, where the changes in CH and CC between time points can be captured with ease. Lodging is influenced by stem strength as well as the weight of the developing pods, and can be defined as a change in the vertical height of plants producing its inclination [45] and can be estimated based on decrease in CH and increase in CC (Figure 10) across time points. Lodging assessments based on the detection of changes in CH between dates 1 to 2 and 1 to 3, correlated with ground truth lodging observations with *r* of 0.58 and 0.57, respectively. Furthermore, including the absolute CC value for the lodging estimation between dates 1 and 3 (Equation (7)), the correlation coefficient increased up to 0.70.

### 3.8. Comparison with Satellite Data

The GRVI data extracted from the satellite images were not significantly correlated with AGBM and seed yield in experiments 1821 and 1821cc (Table 9). In experiments 1822 and 1823, the correlations between GRVI and NDVI mean, and SY were significant but lower than those using UAS data. The lower spatial resolution of the satellite image and spectral mixing (canopy and soil) on the field edges resulted in a poor relationship between image features and ground-reference data. 

**Figure 11:**
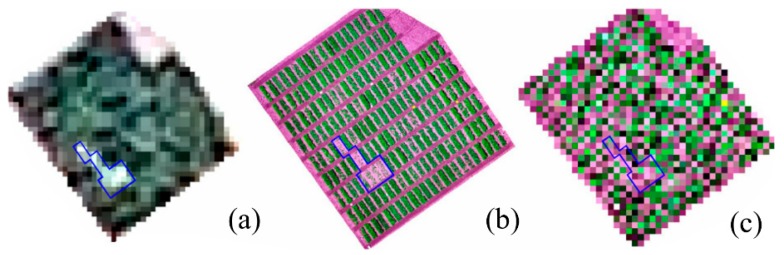
Comparison between the (**a**) RGB satellite image (1.50 m GSD) with (**b**) UAS original image and (**c**) UAS image resampled at a pixel size of 1.50 m GSD. The blue polygon highlights plots with low crop cover that can be identified in the satellite image but not in the UAS resampled image.

The decrease in the satellite image resolution was not directly proportional to the decrease on its relationship with ground reference data. As an example, decreasing the resolution of an UAS image, by resampling its pixel size with the nearest neighbor method, to the level of the satellite image [46] SPOT 6 (1.50 m), resulted in an image in which the field edge was un-recognizable. At the same resolution, the satellite image provided more details of the field (Figure 11), which could be the reason for the significant correlations between satellite image features and seed yield obtained with the 1.5 m satellite image resolution.

In the future, satellite data is anticipated to have higher spectral and spatial resolution. The actively sensed data from orbital Synthetic Aperture Radar sensors offers new opportunities for plant phenotyping [47], because of its feasibility for crop phenology monitoring [48,49], crop height [50], and lodging estimations [51]. Furthermore, the anticipated launch of the FLuorescence EXplorer satellite mission, will provide sun-induced crop fluorescence spectral data that will create new research opportunities [52,53].

## 4. Conclusions

In this study, the potential of UAS-based imaging techniques to estimate biomass and crop performance in pea breeding programs was evaluated. In winter pea experiments, a strong correlation between all the image features and seed yield was observed at flowering; while at pod development and maturity, an increase in the correlations with phenological traits was detected. Spectral data was also found to be useful in leaf type identification. Overall, elevation based remote sensing data was highly correlated with CH_GT_ and was also suitable for lodging assessment in spring pea; furthermore, in some winter pea experiments, this type of information was correlated with F50, FN, and SY. These results were obtained regardless of the use of flight plans with double grid pattern and high overlap percentage.

AGBM was found to be highly correlated with image features at 1268 and 1231 ADD (flowering) in the winter and spring peas, respectively. The Lasso model developed with selected image features was able to estimate AGBM with a high level of accuracy. The proposed methods and feature extraction can be used for evaluating biomass in forage breeding trials as well. The satellite imagery needs to be further explored for phenotyping applications.

## Figures and Tables

**Table 1:** Horizontal and vertical dilutions of precision that estimate GCPs position accuracy on winter and spring pea sites.

GCP	Winter Pea		Spring Pea	
	HDOP (m)	VDOP (m)	HDOP (m)	VDOP (m)
**1**	0.005	0.006	0.010	0.010
**2**	0.005	0.010	0.018	0.011
**3**	0.009	0.008	0.008	0.014
**4**	0.007	0.007	0.008	0.009
**5**	0.007	0.006	0.009	0.011
**6**	0.005	0.001	0.006	0.010
**7**	0.008	0.006	0.005	0.009
**8**	0.008	0.002	0.011	0.008
**9**	0.007	0.011	0.007	0.011
**10**	0.007	0.007	0.008	0.008

**Table 2:** Horizontal and vertical positioning errors from RGB and multispectral imageries (with its respective flight parameters differences) with and without RTK rectification, based on 1268 ADD time point data.

Mosaic	Flight Parameters		Without RTK Rectification		With RTK Rectification	
	Grid Pattern	Overlap (%)	HPE (m)	VPE (m)	HPE (m)	VPE (m)
**RGB**	Double	90–90	2.051	0.819	0.028	0.050
**Multispectral**	Single	80–70	1.834	0.633	0.048	0.079

**Table 3:** Correlation between CH_GT_ and CH_UAS_ for the CSMs obtained from RGB and multispectral imageries (with its respective flight parameters differences) with and without RTK rectification, based on 1268 ADD time point data. All correlation coefficients were significant at *P* < 0.001.

Mosaic	Flight Parameters		Without RTK	With RTK
	Grid Pattern	Overlap (%)	*r* (CH_GT_ & CH_UAS_)	*r* (CH_GT_ & CH_UAS_)
**RGB**	Double	90–90	0.93	0.97
**Multispectral**	Single	80–70	0.91	0.96

**Table 4:** Correlation coefficients (r) between VIs, CH_UAS_, CC, and PV with plant features (F50, PM, FN, and SY) in winter pea experiments 1821, 1821cc, 1822, and 1823.

ADD	Image Feature		1821 (*n* = 10)	1821cc (*n* = 5)	1822 (*n* = 20)				1823 (*n* = 20)			
			SY	SY	F50	PM	FN	SY	F50	PM	FN	SY
**365**	**GRVI**	**Sum**	−0.07	0.65	0.15	−0.23	0.18	0.71 ***	0.47 *	0.43	0.25	0.47 *
		**Mean**	−0.32	0.66	0.07	−0.27	0.07	0.59 **	0.24	0.32	0.20	0.15
	**NDVI**	**Sum**	0.26	0.64	0.20	−0.23	0.34	0.82 ***	0.55 *	0.45 *	0.24	0.60 **
		**Mean**	0.10	0.42	0.20	−0.27	0.34	0.81 ***	0.48 *	0.42	0.20	0.54 *
	**NDRE**	**Sum**	0.35	0.60	0.21	−0.21	0.36	0.81 ***	0.61 **	0.48 *	0.31	0.67 **
		**Mean**	−0.22	0.64	0.26	−0.21	0.30	0.67 **	0.57 **	0.59 **	0.44	0.51 *
	**CH_UAS_**		0.12	0.45	−0.09	−0.48 *	−0.06	0.45 *	0.12	0.05	0.01	0.09
	**CC**		0.19	0.65	0.14	−0.26	0.26	0.72 ***	0.40	0.35	0.25	0.42
	**PV**		0.15	0.46	−0.06	−0.34	−0.04	0.46 *	0.29	0.26	0.17	0.29
**784**	**GRVI**	**Sum**	−0.34	0.83	0.33	−0.08	0.51 *	0.90 ***	0.66 **	0.59 **	0.44	0.68 **
		**Mean**	−0.60	0.62	0.32	−0.07	0.52 *	0.89 ***	0.58 **	0.63 **	0.43	0.59 **
	**NDVI**	**Sum**	0.20	0.40	0.02	0.11	−0.22	−0.16	−0.10	−0.19	−0.26	0.12
		**Mean**	−0.18	0.22	0.04	0.18	−0.11	−0.05	−0.34	−0.16	−0.26	0.05
	**NDRE**	**Sum**	0.31	0.64	0.37	0.02	0.57 **	0.87 ***	0.73 ***	0.59 **	0.42	0.76 ***
		**Mean**	−0.36	−0.08	0.08	0.07	0.48 *	0.66 **	0.52 *	0.67 **	0.27	0.68 ***
	**CH_UAS_**		−0.05	0.52	0.09	−0.25	0.32	0.71 ***	0.18	0.34	0.23	0.36
	**CC**		0.38	0.86	0.41	−0.12	0.54 *	0.94 ***	0.67 **	0.53 *	0.44	0.80 ***
	**PV**		0.06	0.68	0.13	−0.19	0.35	0.76 ***	0.38	0.41	0.28	0.57 **
**1268**	**GRVI**	**Sum**	−0.56	0.83	0.34	0.08	0.59 **	0.91 ***	0.75 ***	0.73 ***	0.56 *	0.83 ***
		**Mean**	−0.75 *	0.68	0.33	0.09	0.56 *	0.90 ***	0.63 **	0.77 ***	0.54 *	0.75 ***
	**NDVI**	**Sum**	−0.07	0.92 *	0.46 *	0.09	0.67 **	0.95 ***	0.74 ***	0.74 ***	0.59 **	0.87 ***
		**Mean**	−0.16	0.69	0.42	0.16	0.62 **	0.92 ***	0.68 **	0.78 ***	0.52 *	0.85 ***
	**NDRE**	**Sum**	0.59	0.85	0.54 *	0.14	0.68 **	0.94 ***	0.77 ***	0.68 ***	0.53 *	0.91 ***
		**Mean**	0.60	0.32	0.60 **	0.10	0.65 **	0.87 ***	0.76 ***	0.71 ***	0.41	0.88 ***
	**CH_UAS_**		0.47	−0.29	0.52 *	0.02	0.58 **	0.88 ***	0.69 ***	0.83 ***	0.65 **	0.78 ***
	**CC**		0.59	0.98 **	0.53 *	0.05	0.68 **	0.98 ***	0.71 ***	0.71 ***	0.63 **	0.84 ***
	**PV**		0.51	0.42	0.52 *	0.03	0.59 **	0.92 ***	0.76 ***	0.78 ***	0.62 **	0.82 ***
**1725**	**GRVI**	**Sum**	0.50	0.78	0.57 **	0.31	0.71 ***	0.87 ***	0.77 ***	0.83 ***	0.52 *	0.79 ***
		**Mean**	0.27	−0.65	0.56 *	0.41	0.69 ***	0.81 ***	0.75 ***	0.85 ***	0.54 *	0.78 ***
	**NDVI**	**Sum**	0.56	0.90 *	0.63 **	0.35	0.77 ***	0.88 ***	0.78 ***	0.87 ***	0.57 **	0.80 ***
		**Mean**	0.38	−0.31	0.63 **	0.41	0.75 ***	0.79 ***	0.79 ***	0.88 ***	0.58 **	0.70 ***
	**NDRE**	**Sum**	0.35	0.07	0.69 ***	0.43	0.73 ***	0.54 *	0.79 ***	0.81 ***	0.54 *	0.53 *
		**Mean**	−0.22	−0.59	0.62 **	0.50 *	0.68 ***	0.25	0.74 ***	0.74 ***	0.50 *	0.41
	**CH_UAS_**		0.70 *	−0.65	0.79 ***	0.13	0.61 **	0.76 ***	0.80 ***	0.73 ***	0.76 ***	0.71 ***
	**CC**		0.67 *	0.90 *	0.67 **	0.12	0.69 ***	0.91 ***	0.71 ***	0.76 ***	0.60 **	0.82 ***
	**PV**		0.67 *	−0.50	0.77 ***	0.14	0.61 **	0.74 ***	0.85 ***	0.72 ***	0.70 ***	0.74 ***
**1948**	**GRVI**	**Sum**	−0.07	−0.44	0.60 **	0.58 **	0.61 **	0.14	0.70 ***	0.74 ***	0.47 *	0.30
		**Mean**	−0.15	−0.17	0.33	0.75 ***	0.51 *	0.08	0.66 **	0.87 ***	0.72 ***	0.47 *
	**NDVI**	**Sum**	0.52	0.44	0.70 ***	0.45 *	0.76 ***	0.63 **	0.80 ***	0.87 ***	0.56 *	0.59 **
		**Mean**	0.20	−0.41	0.62 **	0.58 **	0.61 **	0.20	0.76 ***	0.84 ***	0.59 **	0.37
	**NDRE**	**Sum**	0.30	0.60	0.32	0.25	0.12	−0.15	0.71 ***	0.84 ***	0.61 **	0.35
		**Mean**	−0.11	0.56	−0.04	−0.18	−0.29	−0.33	0.06	0.24	0.33	−0.15
	**CH_UAS_**		0.58	−0.13	0.62 **	−0.02	0.49 *	0.74 ***	0.55 *	0.48 *	0.71 ***	0.54 *
	**CC**		0.61	0.97 **	0.67 **	0.16	0.71 ***	0.87 ***	0.69 ***	0.75 ***	0.65 **	0.79 ***
	**PV**		0.55	0.88 *	0.64 **	0.00	0.52 *	0.77 ***	0.67 **	0.54 *	0.70 ***	0.64 **

* Significant at the 0.05 probability level; ** Significant at the 0.01 probability level; *** Significant at the 0.001 probability level.

**Table 5:** Correlation coefficient between NIRv and NDVI-sum with SY and AGBM obtained at 365 ADD and 1268 ADD.

Feature	Experiment	365 ADD		1268 ADD	
		NIRv	NDVI–Sum	NIRv	NDVI–Sum
**SY**	**1821**	0.53	0.26	−0.22	−0.07
	**1821cc**	0.97 **	0.64	0.79	0.92 *
	**1822**	0.67 **	0.82 ***	0.92 ***	0.95 ***
	**1823**	0.76 ***	0.60 **	0.87 ***	0.87 ***
**AGBM**	**1821**	0.78 **	0.75 *	0.75 **	0.77 **
	**1821cc**	0.96 *	0.75	0.88 *	0.94 *

* Significant at the 0.05 probability level; ** Significant at the 0.01 probability level; *** Significant at the 0.001 probability level.

**Table 6:** Spring pea correlation coefficients (*r*) between VIs, CH_UAS_, and PV with CH_GT_.

ADD	Image Feature	GRVI		NDVI		NDRE		CH_UAS_	CC	PV
		Sum	Mean	Sum	Mean	Sum	Mean			
**1231 (*n* = 159)**	**CH_GT_**	0.69 **	0.75 ***	0.49 *	0.73 ***	0.42	0.61 **	0.80 ***	0.26	0.83 ***
**1424 (*n* = 128)**	**CH_GT_**	0.31	0.29	0.18	0.20	0.16	0.10	0.20	0.16	0.28
**1648 (*n* = 32)**	**CH_GT_**	0.64 ***	0.55 **	0.54 **	0.41 *	0.05	0.14	0.77 ***	0.08	0.67 ***

* Significant at the 0.05 probability level; ** Significant at the 0.01 probability level; *** Significant at the 0.001 probability level.

**Table 7:** Correlation coefficient (*r*) (with its respective n) between VIs, CH_UAS_, and PV with AGBM in the winter (1268 ADD only) and spring pea experiments.

Crop Season		GRVI		NDVI		NDRE		CH_UAS_	CC	PV
		Sum	Mean	Sum	Mean	Sum	Mean			
**Winter**	**Exp. 1821 (*n* = 10)**	0.40	0.22	0.77 **	0.82 **	0.74 *	0.70 *	0.34	0.60	0.57
	**Exp. 1821cc (*n* = 5)**	0.94 *	0.86	0.94 *	0.85	0.96 *	0.64	−0.39	0.48	0.33
**Spring**	**1231 ADD (*n* = 159)**	0.84 ***	0.71 ***	0.77 ***	0.70 ***	0.74 ***	0.66 ***	0.68 ***	0.56 ***	0.81 ***
	**1424 ADD (*n* = 128)**	0.82 ***	0.50 ***	0.64 ***	0.29 ***	0.72 ***	0.51 ***	0.44 ***	0.59 ***	0.77 ***
	**1648 ADD (*n* = 32)**	0.54 **	0.85 ***	0.38 *	0.83 ***	0.35	0.79 ***	0.43 *	0.64 ***	0.77 ***

* Significant at the 0.05 probability level; ** Significant at the 0.01 probability level; *** Significant at the 0.001 probability level.

**Table 8:** Correlation coefficients (*r*) between GRVI-sum with AGBM by leaf type (*af* and *Af*) in spring peas at 1231 ADD. All correlations were significant at *P* < 0.001.

Entries	AGBM
All Plots (*n* = 159)	0.84
*af* (*n* = 10)	0.89
*Af* (*n* = 149)	0.86

**Table 9:** Correlation coefficients (*r*) between UAS-based (1268 ADD) and satellite-based (887 ADD) GRVI and NDVI (sum and mean) with AGBM and SY in the winter pea experiments.

Plant Feature	Experiment	Source	GRVI-Sum	NDVI-Sum	GRVI-Mean	NDVI-Mean
**AGBM**	**1821 (*n* = 10)**	UAS	0.40	0.77 *	0.22	0.82 **
		Satellite	−0.12	−0.21	0.43	0.07
	**1821cc (*n* = 5)**	UAS	0.94 *	0.94 *	0.86	0.85
		Satellite	−0.11	0.21	0.44	0.26
**SY**	**1821 (*n* = 10)**	UAS	−0.56	−0.07	−0.75 *	−0.16
		Satellite	−0.56	0.06	0.35	0.56
	**1821cc (*n* = 5)**	UAS	0.83	0.92 *	0.68	0.69
		Satellite	0.63	0.12	0.81	0.03
	**1822 (*n* = 20)**	UAS	0.91 ***	0.95 ***	0.90 ***	0.92 ***
		Satellite	0.39	0.29	0.63 **	0.46 *
	**1823 (*n* = 20)**	UAS	0.83 ***	0.87 ***	0.75 ***	0.85 ***
		Satellite	−0.26	−0.35	0.46 *	−0.16

* Significant at the 0.05 probability level; ** Significant at the 0.01 probability level; *** Significant at the 0.001 probability level.

## Abbreviations

ADD	accumulated degree days
*Af*	normal leaf type
*af*	semi-leafless leaf type
AGBM	above ground biomass
AGL	above ground level
CC	canopy cover
CH_GT_	ground truth canopy height
CH_UAS_	canopy height estimated from unmanned aerial system
CSM	crop surface model
DSM	digital surface model
DTM	digital terrain model
F50	days to 50% flowering
FN	first flowering node
GCP	ground control point
GRVI	green red vegetation index
GSD	ground sample distance
HDOP	horizontal dilutions of precision
HPE	horizontal positioning error
Lasso	least absolute shrinkage and selection operator
NDRE	normalized difference red edge index
NDVI	normalized difference vegetation index
NE	North direction error
NIR	near infrared
NIRv	NIR vegetation reflectance indicator
PM	days to physiological maturity
PV	plot volume
RE	red edge
RGB image	red-green-blue (digital camera) image
RTK	real-time kinematic
SY	seed yield
UAS	unmanned aerial system
USDA	United States Department of Agriculture
VDOP	vertical dilutions of precision
VI	vegetation index
VPE	vertical positioning error
ΔZNR	elevation differences from non-rectified images
ΔZR	elevation differences from rectified images
